# Synthesis of Controlled-Size Silica Nanoparticles from Sodium Metasilicate and the Effect of the Addition of PEG in the Size Distribution

**DOI:** 10.3390/ma11040510

**Published:** 2018-03-28

**Authors:** Christian Chapa-González, Ana Laura Piñón-Urbina, Perla Elvia García-Casillas

**Affiliations:** Instituto de Ingeniería y Tecnología, Universidad Autónoma de Ciudad Juárez, Av. del Charro 610, Col. Partido Romero, Cd. Juárez, CP 32310 Chihuahua, Mexico; analaura.muu@gmail.com (A.L.P.-U.); pegarcia@uacj.mx (P.E.G.-C.)

**Keywords:** silica nanoparticles, poly(ethylene glycol), ORMOSIL, biomedical applications, sodium metasilicate

## Abstract

Silica nanoparticles are widely studied in emerging areas of nanomedicine because they are biocompatible, and their surface can be modified to provide functionalization. The size is intrinsically related to the performance of the silica nanoparticles; therefore, it is important to have control over the size. However, the silica nanoparticles obtained from sodium metasilicate are less studied than those obtained from tetraethyl orthosilicate. Moreover, the methods of surface modification involve several steps after the synthesis. In this work, the effect of different concentrations of sodium metasilicate on the size of silica nanoparticles was studied. In the same way, we studied the synthesis of organically modified silica nanoparticles in a one-step method, using poly(ethylene glycol). The nanoparticles were characterized by scanning electron microscopy, Fourier-transform infrared spectroscopy, and thermogravimetric analysis. It was found that the size distribution of the silica nanoparticles could be modified by changing the initial concentration of sodium metasilicate. The one-step surface-modification method caused a significant decrease in size distribution.

## 1. Introduction

Silica nanoparticles (Si-NPs) have a promising role in emerging nanomedicine because of their low cytotoxicity, ultra-fine size range (below 100 nm), and they can be modified with molecules of biomedical interest. Its structure is formed along a set of Si–O–Si bonds and their surface is coated by hydroxyl groups, (silanol, Si-OH) [1,2]. In the same way, organic modified silica nanoparticles (ORMOSIL-NPs) are a type of silica-based nanoparticles [3], which by combining organic molecules and functional groups in their structure they acquire additional functions, so they widely extend their in vitro applications to bioimaging [4,5,6], biosensors [7,8], and in vivo applications, such as cancer therapy [9,10,11,12] and drug administration [13,14,15]. The size of the nanoparticles is closely related to the correct performance in the biomedical applications.

Among others physicochemical properties such as pH and zeta potential, size distribution is a determining property in the performance of surface-engineered nanoparticles. Thus, the size of ORMOSIL-NPs can affect both the amount of immobilized drug, as well as the release kinetics. Meanwhile, when modifying the Si-NPs organically, meaning by adding functional groups or polymers, the rate of drug release is directly affected by increasing the diffusion resistance thereof. In the work presented by [16] paclitaxel was loaded to CTAB-ORMOSIL-NPs (85.4 ± 8.4 nm) with a drug loading efficiency of 8.74 ± 1.32% and the accumulative released percentage of paclitaxel was less than 7.5% at neutral pH after 72 h, however, the release percentage were 53.9% at pH 5.0 after 72 h. While in [17] the authors obtained a percentage of paclitaxel encapsulation of 25.6% using paclitaxel-loaded Si-NPs ~100 nm in diameter and reached 82% of drug release in about 172 h (pH 7.4). The findings described in [18] indicated that nanoparticle-based oral drug delivery can be potentially improved by adjusting surface modification and size of the Si-NPs.

On the other hand, the size of the nanoparticles is even related to cellular uptake and cytotoxicity. The cellular uptake efficiency of Si-NPs is size-dependent in the order of 55.6 > 167.8 > 307.6 nm according to [19]. In the same way, a predictive model for cytotoxicity of 20 and 50 nm Si-NPs based on functions of size, concentration and exposure time is proposed in [20]. Also, the potential of Si-NPs to induce the apoptosis of human umbilical vein endothelial cells in culture was investigated by [21], they concluded that cytotoxicity of Si-NPs is closely linked to their size and dose. 

Though in the last decade there is a massive of papers reporting the biomedical application of Si-NPs and ORMOSIL-NPs the fundamental study on the size controlling has been rarely presented. In that sense, it is fundamental to control of the properties of the size distribution and amount of coating of Si-NPs to improve their performance as an agent of nanomedicine. In the literature there are some attempts to control the size of the Si-NPs mainly emphasized on controlling the surfactant and tetraethyl orthosilicate (TEOS) concentrations [22]. However, in this work we focus on adapting the concentration variable of sodium metasilicate in the sol-gel method to obtain a controlled route for modulating the size distribution of Si-NPs and ORMOSIL-NPs. Poly(ethylene glycol) (PEG) was chosen to form the ORMOSIL nanoparticles. PEG is one of the main coatings that is used in nanomedicine for both in vitro and in vivo applications [23,24] because of its effectiveness to inhibit the absorption of proteins in the blood and the capture of them by phagocytic cells. The effect on the size of ORMOSIL on amount of coating were also observed.

## 2. Materials and Methods

The silica nanoparticles with different sizes were prepared by varying the concentrations sodium metasilicate (Na_2_SiO_3_, Thermo Fisher Scientific, Pittsburgh, PA, USA). From an initial 0.10 M Na_2_SiO_3_, four solutions with different concentrations were prepared in a logarithmic relationship. The final concentrations of the dilutions were: 0.01 M, 1.00 mM, 0.10 mM, and 0.01 mM. In each separated experiment, the Na_2_SiO_3_ solution was maintained under magnetic agitation and heated to 80 °C. Then, HCl 0.1 M was added dropwise until pH 6.0. After that, multiple washing was carried out using ethanol and water to remove the formed sodium salts, the obtained precipitated was repeatedly (4 times) collected by centrifugation (10 min, 9000 G). The product was left at 50 °C in the oven for 24 h. After drying time, the samples were placed in vials for further modification and characterization.

PEG (average Mn 400) was supplied by Sigma-Aldrich (St. Louis, MO, USA) and used without further purification. The ORMOSIL-NPs were obtained using the method described above with the condition that PEG was added into the reaction mixture before the addition of HCl. PEG/Na_2_SiO_3_ mass ratio used for ORMOSIL-NPs synthesis was 2/1 in each of the five concentrations of Na_2_SiO_3_ used. Afterward, the product was collected by centrifugation then washed and dried. After drying, the samples were placed in vials for further characterizations.

The morphology and size distribution of the Si-NPs and ORMOSIL-NPs were evaluated by field emission scanning electron microscopy (FE-SEM) using a JEOL JSM-7000f (JEOL México SA de CV, Mexico City, Mexico) equipment operating at 15 kV. The samples were directly dispersed on a doubled-face carbon conductive tape before FE-SEM observation. Images were analyzed using the Scandium software (Olympus, ResAlta Research Tecnologies, Golden, CO, USA, 2010) and diameter of 100 individual nanoparticles were measured directly from the images using the line tool to provide reasonable measures for the size distribution of all samples. The results are presented as mean ± SD, and statistical comparisons between groups were carried out using one-way ANOVA followed by the Student’s *t*-test.

Infrared spectra were obtained by using Fourier Transform Infrared (FTIR) with attenuated total reflection (ATR) spectrometer (Nicolet 6700/Thermo Electron Scientific Instruments Corporation, Madison, WI, USA). Germanium (Ge) crystal was used in ATR measurement due to it high refractive index. The infrared spectra were recorded with a resolution of 4 cm^−1^, and the scan range was set from 4000 to 600 cm^−1^. The results are presented as the average of 32 scans

## 3. Results and Discussion

**Figure 1:**
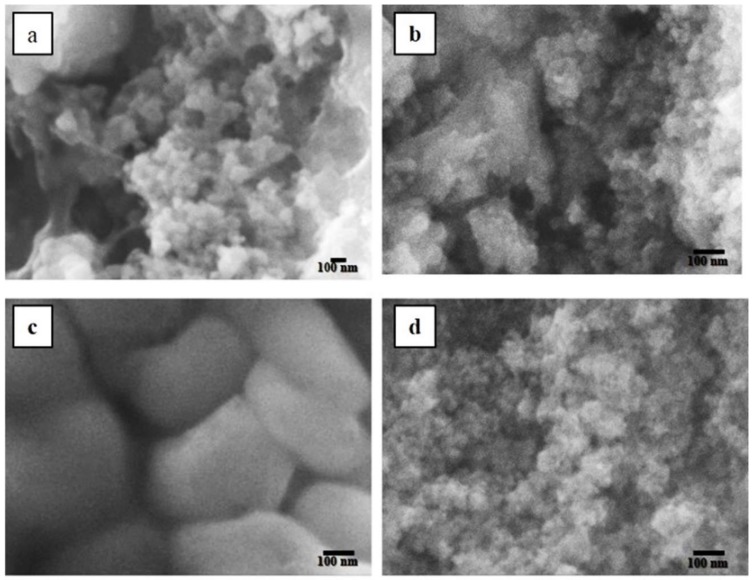
SEM (Scanning Electron Microscopy) images of the materials obtained from different concentrations of Na_2_SiO_3_. (**a**) Si-NPs (Silica Nanoparticles) 0.01 mM; (**b**) ORMOSIL-NPs (Organic Modified Silica Nanoparticles) 0.01 mM; (**c**) Si-NPs 0.10 M; (**d**) ORMOSIL-NPs 0.10 M. In obtaining ORMOSIL-NPs, a 2:1 mass ratio between PEG and Na_2_SiO_3_ was used.

The silicon content increase resulted in the formation of larger particles in the as-synthesized Si-NPs samples and ORMOSIL-NPs due to the silicate ion availability of the for the growth of the particles. Whereas the morphology of the nanoparticles was round to oval and was the size was affected by the modification process. The size of the ORMOSIL-NPs is lower compared to the Si-NPs in all the samples, it was clearly evidenced by SEM (Figure 1). Si-NPs obtained from Na_2_SiO_3_ 0.01 mM (Figure 1a) had a size of ~35 nm while ORMOSIL-NPs obtained from Na_2_SiO_3_ 0.01 mM (Figure 1b) had a size of ~17 nm. The variation in size by the effect of PEG is more evident in materials obtained from Na_2_SiO_3_ 0.1 M. The size of Si-NP (Figure 1c) was ~250 nm and the size of ORMOSIL-NPs (Figure 1d) was ~60 nm. The size distribution of the nanoparticles in the Si-NPs were higher than ORMOSIL-NPs in all the samples obtained from the solution with the same concentration of the precursor. The considerable decrease in the sizes of the coated nanoparticles in relation to the uncoated ones, since PEG acts as a surfactant that prevents the nanoparticles from agglomerating. According to [1] the surfactant is one of the main factors influencing the material structuring. Since PEG acts as both, coating and surfactant, its concentration and the molar ratio between the surfactant and the silica source caused a decrease in the final size of the ORMOSIL-NPs.

**Figure 2:**
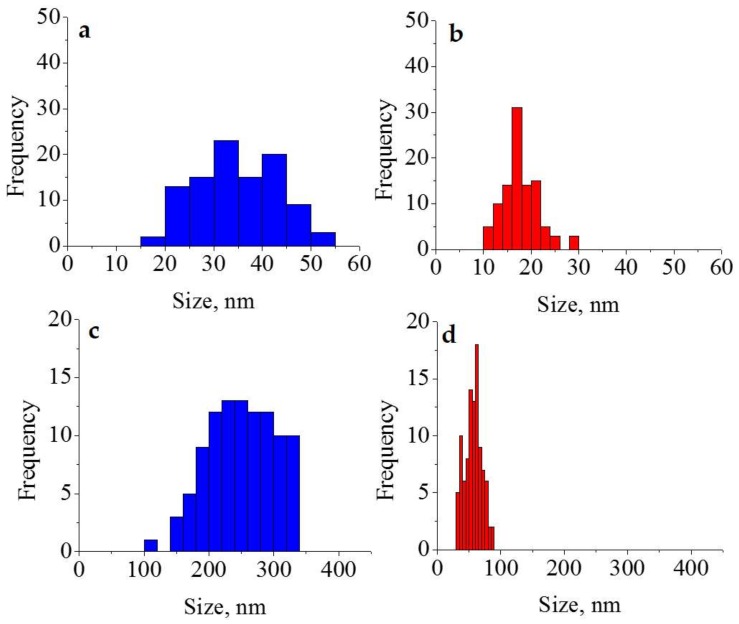
Histograms generated from the measurement of particle size (*n* = 100). (**a**) Si-NPs 0.01 mM; (**b**) ORMOSIL-NPs 0.01 mM; (**c**) Si-NPs 0.10 M; (**d**) ORMOSIL-NPs 0.10 M.

The histogram of the size distribution of the Si-NPs and ORMOSIL-NPs, obtained with the lowest concentration of Na_2_SiO_3_, can be seen in Figure 2a and Figure 2b respectively. Likewise, the histograms of Si-NPs and ORMOSIL-NPs of the materials obtained at the highest precursor concentration are also shown (Figure 2c and Figure 2d respectively). It can be noted that the size distribution is narrower when the PEG is used, it happened in all cases. Steric repulsion, caused by PEG molecules, restricts the growth of nanoparticles. 

**Figure 3:**
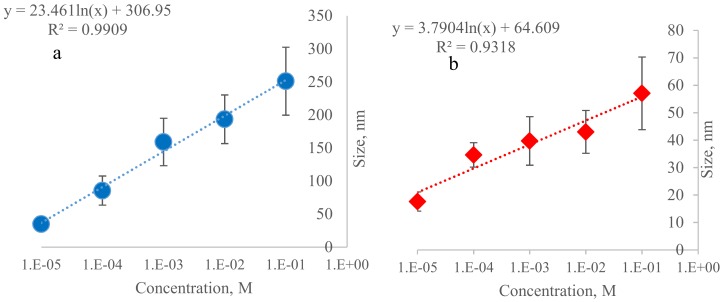
(**a**) Relationship between the initial concentration of Na_2_SiO_3_ and the size distribution of Si-NPs. (**b**) Relationship between the initial concentration of Na_2_SiO_3_ and the size distribution of ORMOSIL-NPs.

In Figure 3a we can see the relationship between the sizes of Si-NPs obtained from the different initial concentrations of Na_2_SiO_3_. A direct relationship can be noted between the average size and concentration. Similarly, Figure 3b shows the relationship between the sizes of ORMOSIL-NPs and the initial concentration of the precursor. The coating with PEG did significantly (*P* > 0.05) alter mean size. In the same way, particle size distributions before and after modification with PEG were significantly different (*P* > 0.05).

**Figure 4:**
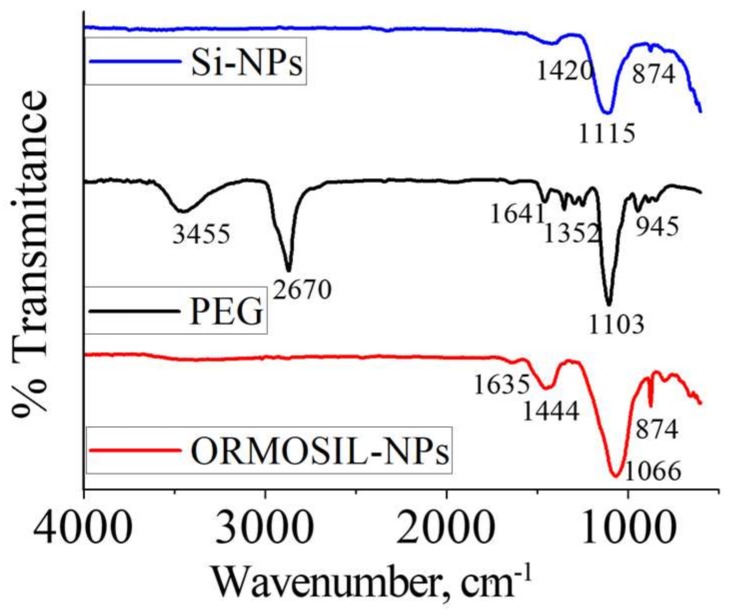
FTIR (Fourier Transform Infrared) spectra of Si-NPs, PEG, and ORMOSIL-NPs.

**Figure 5:**
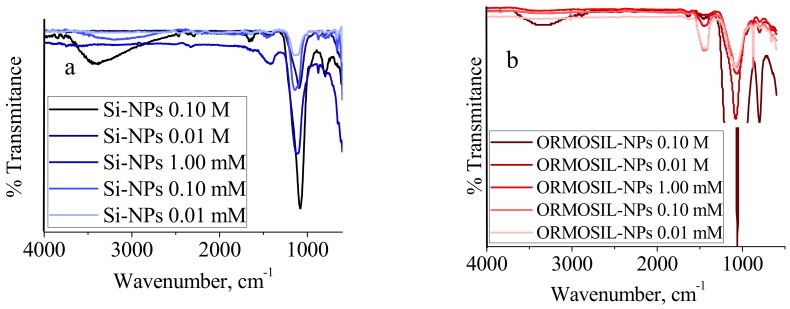
(**a**) FTIR spectra of Si-NPs with decreasing molar concentration of Na_2_SiO_3_ as a precursor. (**b**) FTIR spectra of ORMOSIL-NPs with decreasing molar concentration of Na_2_SiO_3_ as a precursor.

The chemical signature of PEG molecules associated to Si-NPs was demonstrated by IR spectroscopy on dried samples (Figure 4). In Si-NPs the absorption peak at 1110 cm^−1^ is assigned to the Si–O–Si asymmetric stretching vibration, and the peak at 874 cm^−1^ is ascribed to the stretching vibration of Si–OH. The IR spectrum of PEG exhibits a very broad band between 3600 and 3100 cm^−1^ attributed to the stretching vibrations of OH groups. The IR band at 2670 cm^−1^ is attributed to asymmetric CH stretching vibrations. A peak at 1641 cm^−1^ is due to carbonyl group while band at 1352 cm^−1^ was assigned to the symmetrical stretching modes of the alkyl chains (CH_2_). In comparison with bare Si-NPs, the ORMOSIL-NPs IR spectra show new absorption peaks at 1635 and 1350 cm^−1^, corresponding to the stretching vibration of alkyl groups. Moreover, in ORMOSIL-NPs appears a shoulder at ~1100 cm^−1^ that may be attributed to stretching vibrations of C-O-C ether bonds. Comparison of the Si-NPs samples in Figure 5a confirms that by decreasing Na_2_SiO_3_ concentration, the intensity of the peaks related to Si-O-Si characteristic peaks are more enhanced. Furthermore, in the ORMOSIL-NPs spectra (Figure 5b), the characteristic bands of the silica are observed and the shoulder at 1100 cm^−1^ described above, as well as peaks attributed to the alkyl chains.

**Figure 6:**
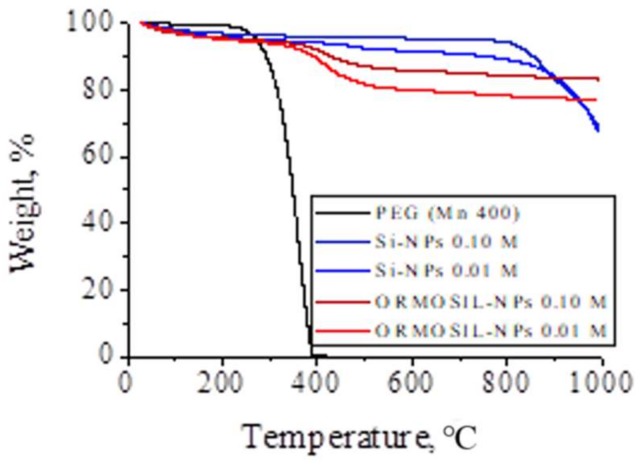
Mass percent versus temperature thermogram of PEG (Mn 400), Si-NPs and ORMOSIL-NPs

Thermogravimetric analysis (TGA) diagrams (Figure 6) of PEG, Si-NPs (0.10 M and 0.01 M), ORMOSIL-NPs (0.10 M and 0.01 M) samples were performed in the same conditions for a better comparison. The complete thermal decomposition of PEG started at 250 °C and ended at ~400 °C, the diagram shows the uniform decomposition in just one step without intermediate stages. This is due to the PEG has a simple structure of linear chain. The TGA diagrams of Si-NPs and ORMOSIL-NPs, obtained from the same initial concentration of Na_2_SiO_3_, show a significant difference of mass losses between them, from 5.73% for Si-NPs up to 15.73% for ORMOSIL-NPs for the initial Na_2_SiO_3_ 0.10 M and from 10.93% for Si-NPs up to 21.76% for ORMOSIL-NPs for the PEG/Na_2_SiO_3_ 0.01 M. The TGA diagrams of Si-NPs shows a main loss of mass around 100 °C due to the release of water associated to the silica structure. The curves show a gradual decrease from tends to remain constant from 100 °C to 800 °C. Instead, the TGA diagrams of ORMOSIL-NPs the weight loss at temperature lower than 100 °C correspond to the release of absorbed water (~5%). The second weight loss corresponds to the PEG decomposition. The thermal decomposition is delayed approximately 100 °C, in comparison with free PEG. The interaction between PEG and silica causes the degradation temperature to occur at higher temperatures. Note that the minor NPs size is quite effective for immobilizing PEG since the weight amount of the polymer in the ORMOSIL-NPs is about 5% higher. This difference is because smaller NPs have a larger surface area, so that there are more functional groups that can interact with PEG molecules.

## 4. Conclusions

In this work, the effect of sodium metasilicate concentration and PEG/Na_2_SiO_3_ ratio on the synthesized Si-NPs and ORMOSIL-NPs has been investigated. The average diameter size obtained by FE-SEM images demonstrated that the particles size decreases in direct relation with the Na_2_SiO_3_ concentration. The result reported in the present work showed that using PEG in a mass ratio 2:1 caused a significant decrease in size distribution of nanoparticles. Thereby, depending on the reaction conditions, the size of the final material can be tuned. Infrared spectroscopy confirmed the successful modification of Si-NPs. The amount of PEG adsorbed on ORMOSIL-NPs was remarkably increased in the material that has a smaller average size. The results propose an effective method to control the size of the NPs in those biomedical applications where an adequate size is required.
